# Tumor genomic profiling and personalized tracking of circulating tumor DNA in Vietnamese colorectal cancer patients

**DOI:** 10.3389/fonc.2022.1069296

**Published:** 2022-12-12

**Authors:** Huu Thinh Nguyen, Trieu Vu Nguyen, Van-Anh Nguyen Hoang, Duc Huy Tran, Ngoc An Le Trinh, Minh Triet Le, Tuan-Anh Nguyen Tran, Thanh Huyen Pham, Thi Cuc Dinh, Tien Sy Nguyen, Ky Cuong Nguyen The, Hoa Mai, Minh Tuan Chu, Dinh Hoang Pham, Xuan Chi Nguyen, Thien My Ngo Ha, Duy Sinh Nguyen, Du Quyen Nguyen, Y-Thanh Lu, Thanh Thuy Do Thi, Dinh Kiet Truong, Quynh Tho Nguyen, Hoai-Nghia Nguyen, Hoa Giang, Lan N. Tu

**Affiliations:** ^1^ University Medical Center, Ho Chi Minh City, Vietnam; ^2^ Thu Duc City Hospital, Ho Chi Minh City, Vietnam; ^3^ Medical Genetics Institute, Ho Chi Minh City, Vietnam; ^4^ Gene Solutions, Ho Chi Minh City, Vietnam; ^5^ Department of Oncology, Faculty of Medicine, Nguyen Tat Thanh University, Ho Chi Minh City, Vietnam

**Keywords:** mutational landscape, somatic mutation, minimal residual disease (MRD), circulating tumor (ctDNA), next-generation sequencing (NGS)

## Abstract

**Background:**

Colorectal cancer (CRC) is the fifth most common cancer with rising prevalence in Vietnam. However, there is no data about the mutational landscape and actionable alterations in the Vietnamese patients. During post-operative surveillance, clinical tools are limited to stratify risk of recurrence and detect residual disease.

**Method:**

In this prospective multi-center study, 103 CRC patients eligible for curative-intent surgery were recruited. Genomic DNA from tumor tissue and paired white blood cells were sequenced to profile all tumor-derived somatic mutations in 95 cancer-associated genes. Our bioinformatic algorithm identified top mutations unique for individual patient, which were then used to monitor the presence of circulating tumor DNA (ctDNA) in serial plasma samples.

**Results:**

The top mutated genes in our cohort were *APC*, *TP53* and *KRAS*. 41.7% of the patients harbored *KRAS* and *NRAS* mutations predictive of resistance to Cetuximab and Panitumumab respectively; 41.7% had mutations targeted by either approved or experimental drugs. Using a personalized subset of top ranked mutations, we detected ctDNA in 90.5% of the pre-operative plasma samples, whereas carcinoembryonic antigen (CEA) was elevated in only 41.3% of them. Interim analysis after 16-month follow-up revealed post-operative detection of ctDNA in two patients that had recurrence, with the lead time of 4-10.5 months ahead of clinical diagnosis. CEA failed to predict recurrence in both cases.

**Conclusion:**

Our assay showed promising dual clinical utilities in residual cancer surveillance and actionable mutation profiling for targeted therapies in CRC patients. This could lay foundation to empower precision cancer medicine in Vietnam and other developing countries.

## Introduction

Colorectal cancer (CRC) is the third most commonly diagnosed and the second leading cause of cancer death worldwide (1). In Vietnam, CRC accounts for 9.0% of all cancer cases in both women and men, with 16,426 new cases and 8,203 deaths in 2020 (1). Recent advances in next generation sequencing (NGS) have enabled genetic data-driven decision making in clinical oncology. For example, the discovery that *KRAS* mutations are predictive of primary resistance to the EGFR inhibitor Erbitux^®^ has changed the clinical use of this drug for metastatic CRC. In developing countries like Vietnam, however, access to genetic testing is still limited due to high cost and lack of trained laboratories. Therefore, the mutational landscape of CRC in Vietnam and its translational potential for precision medicine are currently unknown.

Together with the rising incidence of CRC, the 5-year survival rate of Vietnamese patients was reported at only 45.0% (2), lower than that in other countries (3, 4). A major cause of cancer death is metastatic recurrence, potentially due to residual cancer cells remaining after curative-intent treatment including surgery and adjuvant therapies. Currently, there are limited clinical tools to help identify patients with post-operative residual disease that may benefit from additional or more intensive systemic therapy. Imaging methods and blood test to detect the biomarker carcinoembryonic antigen (CEA) both have limited sensitivity and specificity to detect residual tumor burden and hence often fail to identify patients at risk for relapse early (5, 6).

Circulating tumor DNA (ctDNA) is a type of cell-free DNA (cfDNA) released from cancer cells into the bloodstream. ctDNA can be distinguished from normal cfDNA based on different alterations such as somatic mutations and epigenetic changes. Several longitudinal clinical trials have demonstrated that residual tumor monitoring by ctDNA in liquid biopsy is effective for many solid tumors particularly CRC. Patients who had post-operative ctDNA positive had a significantly higher risk of recurrence and metastasis compared to those negative for ctDNA (7, 8). In addition to the prognostic value, ctDNA monitoring allowed detection of CRC relapse earlier than conventional methods by an average lead time of 4-10.9 months (3, 7), allowing for opportune intervention to improve overall survival. Currently, ctDNA monitoring technology is only available in developed countries and remains unaffordable for majority of the patients.

With the goal of making precision medicine accessible and affordable to the Vietnamese, we established K-Track^®^, a streamlined and affordable assay with dual clinical utilities in residual cancer surveillance and actionable mutation profiling for targeted therapies. Our interim analysis showed that the assay could stratify patients based on post-treatment ctDNA status and detect relapse early ahead of clinical diagnosis.

## Materials and method

### Patients and sample collection

**Figure 1:**
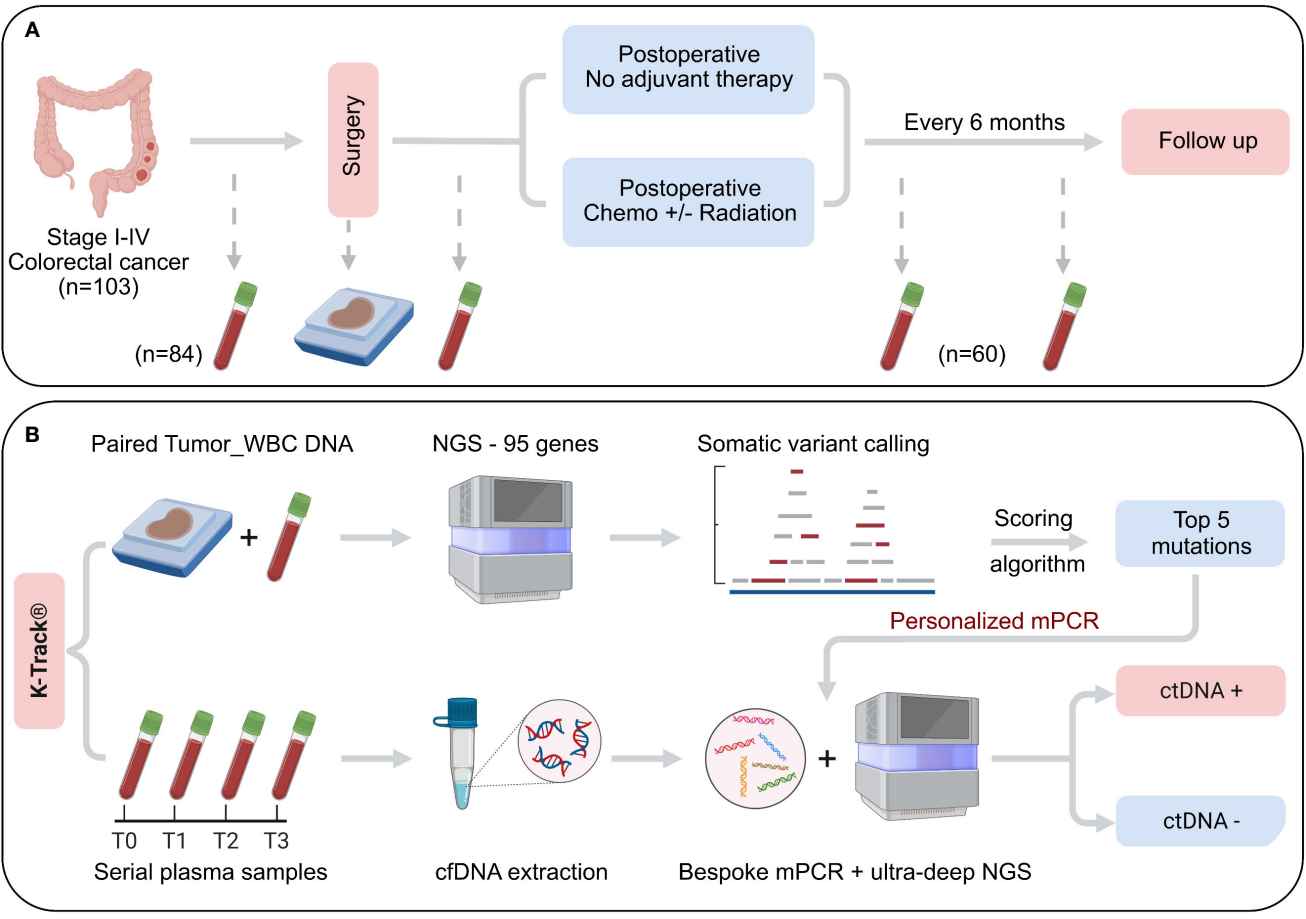
Schematic of study design and K-Track^®^ assay. **(A)** 103 patients with primary colorectal cancer stage I-IV, eligible for curative-intent surgery were enrolled. Serial plasma samples were collected before surgery and at scheduled visits after surgery. FFPE samples of surgically removed tumors were also collected. Clinical outcomes were recorded at each visit. **(B)** Genomic DNA of paired FFPE and WBC were sequenced to profile all tumor-specific somatic alterations in 95 cancer-associated genes. Top 5 mutations were selected by our K-Track^®^ scoring algorithm and then used to monitor ctDNA presence in plasma samples by a bespoke multiplex PCR assay and ultra-deep sequencing at an average of 100,000X.

In this prospective multicenter cohort study, 103 patients diagnosed with stage I-IV CRC were recruited at the University Medical Center, Thu Duc city Hospital, and Medical Genetics Institute in Ho Chi Minh city, Vietnam from April 2021 to June 2022. Patients must be at least 18 years old, eligible for curative-intent surgery and had not received any cancer treatment, or experienced recurrence prior to the time of study entry. 10 mL of peripheral blood was serially collected: less than 14 days before surgery, 30 days after surgery and then at scheduled follow-up visits every 6 months. 6-8 sections of formalin-fixed paraffin-embedded (FFPE) tumor samples with at least 60% tumor cellularity were also collected. CEA level was measured at each visit by the diagnostic laboratory at the participating site and CEA level of less than 5 ng/mL was considered normal. All patients received treatment according to standard-of-care; clinicopathological and treatment information was provided by physicians in a standardized format. Clinical recurrence and/or metastasis was confirmed by either imaging or biopsy result. Patient demographics were listed in **Table 1**; study design and sample analysis workflow were in **Figure 1** (created with BioRender.com).

**Table 1:** Patient demographics.

Characteristic	N = 103
**Median age at diagnosis (range), year**	60 (27 – 85)
**Gender, N (%)**	
Female	45 (43.7)
Male	58 (56.3)
**Size of tumor, mean (range), cm**	4.9 (2 – 15)
**Number of tumors, mean (range)**	1 (1 – 2)
**Tumor site, N (%)**	
*Colon*	*68 (66.0)*
Left	28 (27.2)
Right	23 (22.3)
Transverse	4 (3.9)
Sigmoid	7 (6.8)
Unknown	6 (5.8)
*Rectal*	*26 (25.2)*
*Not available*	*9 (8.8)*
**Clinical nodal status, N (%)**	
Negative	47 (45.6)
Positive	35 (34.0)
Not available	21 (20.4)
**Histological grade, N (%)**	
1	0 (0.0)
2	72 (69.9)
3	8 (7.8)
Not available	23 (22.3)
**TNM stage, N (%)**	
I	13 (12.6)
II	41 (39.9)
III	40 (38.8)
IV	3 (2.9)
Not available	6 (5.8)

All patients provided written informed consent to participate in the study and to the anonymous use of their samples, clinical and genomic data for this study. All genomic data were de-identified and aggregated for the genetic analysis of the cohort.

### Tumor sample processing

Genomic DNA was isolated from FFPE and matching white blood cells (WBC) samples by the QIAamp DNA FFPE Tissue Kit (Qiagen, USA) and the MagMAX™ DNA Multi-Sample Ultra 2.0 kit (ThermoFisher, USA) respectively according to manufacturers’ instructions. 150-200 ng of gDNA was used for library preparation. Specifically, DNA fragmentation and library preparation for both FFPE and WBC samples were performed using the NEBNext Ultra II FS DNA library prep kit (New England Biolabs, USA). Libraries were hybridized with predesigned probes for a gene panel of 95 targeted genes (Integrated DNA Technologies, USA). This panel includes the top 20 most frequently mutated genes in CRC and other solid tumors as reported in the Catalogue of Somatic Mutations in Cancer (COSMIC) database (**Table S1**). DNA libraries were sequenced on the DNBSEQ-G400 sequencer (MGI, China) with an average target coverage of 200X. A sample passed quality control when the percentage of target regions that did not reach coverage = 1 over any base was less than 1% and the percentage of all target bases achieving 20X or greater coverage depth was over 98%.

### Tumor variant calling and ranking

Sequencing data were processed based on best practices workflows from Genome Analysis Tool Kit (GATK) for somatic variant calling (9). First, both read 1 and read 2 in paired-end Fastq files were assessed using FastQC (10) for total number of reads, quality score distribution across all bases, quantification of contaminants, and estimates of duplication rate. Reads were then aligned to the human reference genome (GRCh38) by BWA-MEM (v0.7.15) (11). Post-alignment procedures including sorting, marking duplicated reads and assessing alignment quality was done by Picard (v2.25.6) (12). Somatic variants were called by GATK MuTect2 (v4.0.12.0) (13) in the tumor-normal mode for paired FFPE and WBC samples with the use of a panel of normals and the population allele frequency from The Genome Aggregation Database (gnomAD). This step was to remove sequencing noise, germline variants and clonal hematopoiesis of intermediate potential (CHIP) variants. All filtered variants were further assessed for their functional impact using Variant Effect Predictor with the data from COSMIC and Clinvar databases. For mutational spectrum analysis, a minimum Variant allele frequency (VAF) of 5% in FFPE was applied for additional filtering. The annotated Variant Call Format (VCF) was then converted to the Mutation Annotation File (MAF) format using vcf2maf (doi:10.5281/zenodo.593251). The MAF data were analyzed and visualized by the ‘maftools’ in R package v3.4.2 (14).

All non-synonymous alterations were ranked by our K-Track^®^ scoring algorithm to identify the most potential tumor-derived mutations to track. Ranking criteria include 1) VAF in FFPE; 2) being predicted as pathogenic/deleterious in the Clinvar and COSMIC databases or by SIFT and Polyphen; 3) being a stop-gained mutation in a tumor suppressor gene (by COSMIC classification); 4) being a mutation in an oncogene (by COSMIC classification) with reported frequency of more than 3 times in COSMIC; 5) validated as a tumor-derived mutation in our in-house database. Exclusion criteria included mutations being located in low complexity regions. The top mutations unique to each patient were selected to design bespoke multiplex PCR assays in plasma.

### Plasma sample processing and multiplex PCR

cfDNA was extracted from plasma samples using the MagMAX™ Cell-Free DNA Isolation Kit (ThermoFisher, USA). cfDNA concentration was quantified using the QuantiFluor^®^ dsDNA system (Promega, USA). A concentration of ≥ 0.1 ng/uL or total of ≥ 3 ng of cfDNA was required. An average cfDNA input for mPCR assay was 6.9 ng (range 3-20 ng). Compatible primers were designed by Primer3Plus software and synthesized by PhuSa Biochem, Vietnam. cfDNA fragments carrying the selected mutation sites were amplified in a PCR reaction containing designed primer pairs and enzyme KAPA HiFi DNA Polymerase (Roche, USA). Amplified cfDNA fragments were indexed and sequenced on the NextSeq 2000 system (Illumina, USA) with an average depth of 100,000X per amplicon. Amplicons with less than 10,000X coverage were considered failed.

### Plasma variant calling and ctDNA analysis

The raw fastq data of amplicons were removed adapters with Trimmomatic (v0.39) (15), mapped to the human reference genome (GRCh38) using BWA-MEM (v0.7.15), sorted and marked duplicates using Picard (v2.25.6). Variant calling was performed using mpileup from Samtools (v1.11) (16).

To determine limit of detection (LOD), we used commercial reference standards Tru-Q1 and Tru-Q0 (Horizon Discovery, USA) and titrate the somatic mutations at average VAFs of 3%, 0.5%, 0.1%, 0.05% and 0% based on DNA input. The mixtures were fragmented to mimic cfDNA length and then processed through the mPCR workflow as above. The observed VAF was compared with the expected VAF for each mutation to determine the LOD of the assay. In addition, negative cfDNA samples isolated from 150 plasma samples of healthy donors were also subject to the same workflow to determine the false-positive rate of the assay.

A sample was called positive for ctDNA if at least one tracked mutation was detected with VAF ≥ LOD. Mean VAF of a sample was calculated as mean of all positive mutations if present. If no mutations were found positive, mean VAF was the mean of all tracked mutations.

### Statistical analysis

For continuous variables including the number of mutations, VAF, cfDNA, ctDNA and CEA levels, Mann-Whitney U test was performed for comparison between 2 groups; Kruskal-Wallis with *post hoc* Dunn’s test was performed for more than 2 groups. For the categorical variable of the ctDNA detection rate, Chi-squared test and Fisher’s exact test were used. All statistical tests were performed in Graphpad Prism and considered significant at p < 0.05.

## Results

### Study design and participants

Among 103 Vietnamese CRC patients recruited, the median age of the patients was 60 (range: 27 – 85) years old with a balanced ratio of males (56.3%) and females (43.7%) (**Table 1**). All patients had carcinoma at TNM stage I (12.6%), II (39.9%), III (38.8%), and IV (2.9%). 66.0% of them had colon cancer while 25.2% had rectal cancer. Majority had 1 tumor with an average tumor size of 4.9 cm and intermediate histological grade (69.9%). 34.0% of the cases had spread to lymph nodes (**Table 1**).

In our K-Track^®^ assay, FFPE tumor and serial plasma samples were collected before and after surgery at scheduled visits (**Figure 1A**). FFPE samples were collected for all 103 patients; 84 of them provided pre-operative blood samples and until June 2022, 60 patients had post-operative blood samples collected (**Figure 1A**). Genomic DNA from paired FFPE and WBC were hybridized to the predesigned 95-gene panel to identify all tumor-derived alterations. Our scoring algorithm described in the Method was used to rank and select top mutations for each patient, which were then used to track ctDNA in the plasma. The detection of ctDNA was then compared with clinical outcomes at each visit (**Figure 1B**).

### Mutational landscape

**Figure 2:**
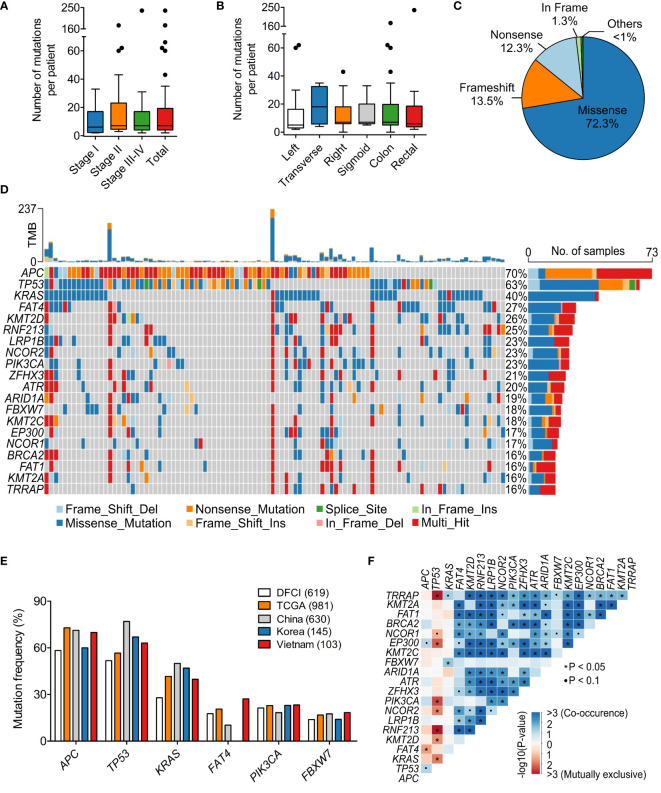
Mutational spectrum of 95 genes in the Vietnamese colorectal cancer patients. **(A)** The average number of tumor-derived mutations was 7 mutations per patient and not different by stage. **(B)** The mutation burden was not different by the tumor site. **(C)** Pie chart showing the distribution of mutation classes identified in 95 genes. **(D)** The top 25 significantly mutated genes in our cohort. **(E)** Mutation frequency of top mutated genes in our cohort was compared with published datasets of Caucasian and Asian cohorts. **(F)** Mutually exclusive and co-occurring mutated genes in our dataset. *P < 0.05; Kruskal-Wallis and *post hoc* Dunn’s test for **(A, B)**.

Sequencing results of paired FFPE-WBC showed that 99.0% of the patients had at least 1 somatic mutation in the 95 examined genes. We observed a wide range of 2 to 237 somatic mutations, with an average of 7 mutations per patient (**Figure 2A**). The mutation burden was not affected by the TNM stage or the tumor site (**Figures 2A, B**). Majority of the mutations were missense (72.3%), followed by frameshift (13.5%) and nonsense (12.3%) mutations (**Figure 2C**).

The most frequently mutated genes in our cohort were *APC* (69.9%), *TP53* (63.1%) and *KRAS* (39.8%) (**Figure 2D**). While missense mutations were dominant for most of the highly mutated genes, *APC* was the exception with primarily nonsense mutations (51.7%) (**Figure 2D**). We then compared the mutation frequency in our cohort with published CRC datasets from the Caucasian cohorts: TCGA (n=981) (17, 18) and DFCI (n=619) (19); as well as the Asian cohorts: China (n=630) (20) and Korea (n=145) (21). The frequency of *TP53* mutations in the Vietnamese seemed to be slightly higher than the Caucasian and more comparable with the Asian (**Figure 2E**). Interestingly, *FAT4* mutations (27.2%) followed the opposite trend that the mutation frequency in the Vietnamese was more similar to the Caucasian, which was twice more prevalent than the Chinese (**Figure 2E**).

When examining the pattern of mutual exclusivity and co-occurrence of all mutations, we found that multiple gene pairs had co-occurring mutations (**Figure 2F**). Mutual exclusivity was less abundant and the most significant mutually exclusive genes were *TP53* with either *TRRAP*, *RNF213*, *KRAS* or *PIK3CA* (**Figure 2F**). Besides, among the 95 examined genes, *KRAS* showed a prominent mutation hotspot at amino acid Glycine 12, as G12D/S/V/C/A mutations accounted for 56.8% of all *KRAS* mutated cases (**Figure S1**).

### Actionable alterations

**Figure 3:**
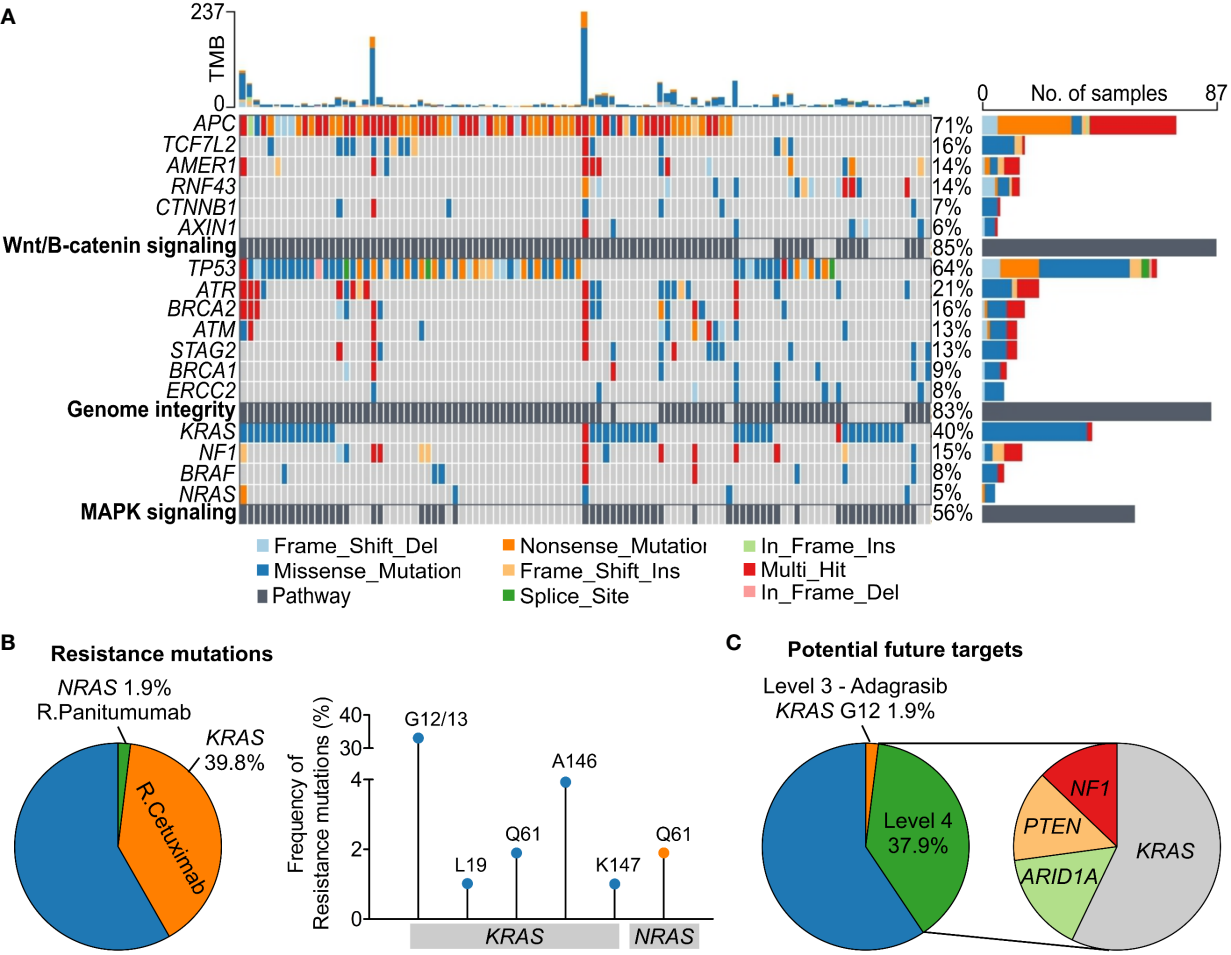
Oncogenic signaling pathways and actionable alterations in the Vietnamese colorectal cancer patients. **(A)** The top three signaling pathways with frequent oncogenic alterations in our cohort were Wnt/β-catenin signaling, genome integrity, and MAPK signaling. **(B)** Proportions of patients harboring mutations in *KRAS* and *NRAS* predictive of resistance to Cetuximab and Panitumumab respectively. Frequency of the specific resistance mutations was also illustrated. **(C)** Proportions of patients carrying mutations that are candidate biomarkers for response to drugs with compelling clinical evidence (level 3) or laboratory evidence (level 4) as classified by the OncoKB database.

The top three signaling pathways being altered in our CRC cohort were Wnt/β-catenin signaling (*APC*, *TCF7L2*, *AMER1*, *RNF43*), genome integrity (*TP53, ATR*), and mitogen-activated protein kinase – MAPK signaling (*KRAS, NF1*) with the mutation frequency of 85.3%, 83.3% and 55.9% respectively (**Figure 3A**). We then characterized actionable alterations in our cohort who might benefit from genetic sequencing. The OncoKB database (22), an expert-curated precision oncology knowledge base, was used to classify somatic alterations with treatment implications stratified by different levels of evidence (22). The list of alterations and corresponding drugs for CRC were listed in **Table S2**. In total, 1.9% of patients had *BRAF* V600E mutation predictive of response to the approved drug Encorafenib. 41.7% of the patients had at least 1 somatic mutation predictive of resistance to the level 1 FDA-approved drugs (**Figure 3B**). Majority (39.8%) of them were *KRAS* resistance mutations to Cetuximab (**Table S2**), with G12/13 being the most common site (33.0%). 1.9% of the patients had *NRAS* Q61 mutation associated with resistance to Panitumumab (**Figure 3B**). Besides FDA-approved drugs, a few experimental drugs have demonstrated therapeutic effects either in clinical studies (level 3 drug - Adagrasib) or biological research (level 4 drugs – **Table S2**) and they might benefit about 1.9% and 37.9% respectively of the Vietnamese CRC patients in the future (**Figure 3C**).

### Personalized tracking of ctDNA in plasma

**Figure 4:**
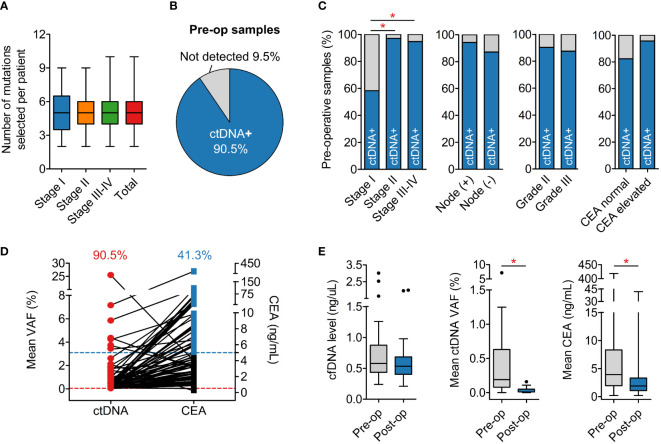
Detection of ctDNA in plasma samples. **(A)** The average number of mutations selected to track was 5 mutations per patient regardless of cancer stage. **(B)** Detection rate of ctDNA in pre-operative plasma samples was 90.5%. **(C)** Pre-operative ctDNA detection rate was associated with TNM stage, as the rate in stage I was significantly lower than in stage II and III. Nodal involvement, histological grade and CEA level status did not affect the detection rate. **(D)** Pre-operative CEA level was found elevated (≥5 ng/mL) in only 41.3% patients. **(E)** Total levels of cfDNA were not different between pre-operative and post-operative plasma samples while ctDNA and CEA levels significantly reduced after surgery. *P < 0.05; Kruskal-Wallis and *post hoc* Dunn’s test for **(A)**; Chi-squared test and Fisher’s exact test for **(C)**; Mann-Whitney U test for **(E)**.

The set of somatic mutations identified in the tumor FFPE was subjected to our developed algorithm for ranking based on several criteria (described in Methods). Those with the highest score and highest VAF in FFPE were selected for tracking. Based on our analysis, VAF of a mutation in FFPE was a critical factor for its likelihood of detection in plasma because mutations with VAF less than 10% in FFPE had a much lower detection rate in plasma compared to those with VAF ≥ 10% (**Figure S2A**). On average, we selected 5 (range 2-10) mutations per patient regardless of the TNM stage (**Figure 4A**).

Personalized multiplex PCR and ultra-deep sequencing were performed to detect ctDNA in plasma samples with an average read depth of 100,000X per amplicon. In this dataset, 3.8% amplicons with less than 10,000X coverage were considered failed and removed from downstream analysis (**Figure S2B**). In our LOD assay, mutations at frequency below 0.05% could still be detected but false-positive signals from healthy plasma samples were also recorded with VAF < 0.05% (**Figure S2C**). Therefore, we chose the cut-off of 0.05% to keep the false-positive rate below 1% (**Figure S2D**). Any mutation with VAF ≥ 0.05% in plasma samples was called “positive”.

The average number of positive mutations detected in the plasma was 2 (range 1-9) mutations per patient, accounting for ≥ 50% of tracked mutations in most cases. A plasma sample was called “positive” for ctDNA when at least 1 tracked mutation was positive. The overall detection rate in pre-operative plasma samples was 90.5% (**Figure 4B**). This rate was found to be associated with the TNM stage as the ctDNA detection rate in stage I cancer was significantly lower than stage II-IV (**Figure 4C**). Other clinicopathological variables such as nodal involvement, tumor histological grade and CEA level status did not affect ctDNA detection (**Figure 4C**). Furthermore, pre-operative CEA measurement showed that only 41.3% of the patients had elevated CEA levels, lower than the ctDNA detection rate (**Figure 4D**).

**Figure 5:**
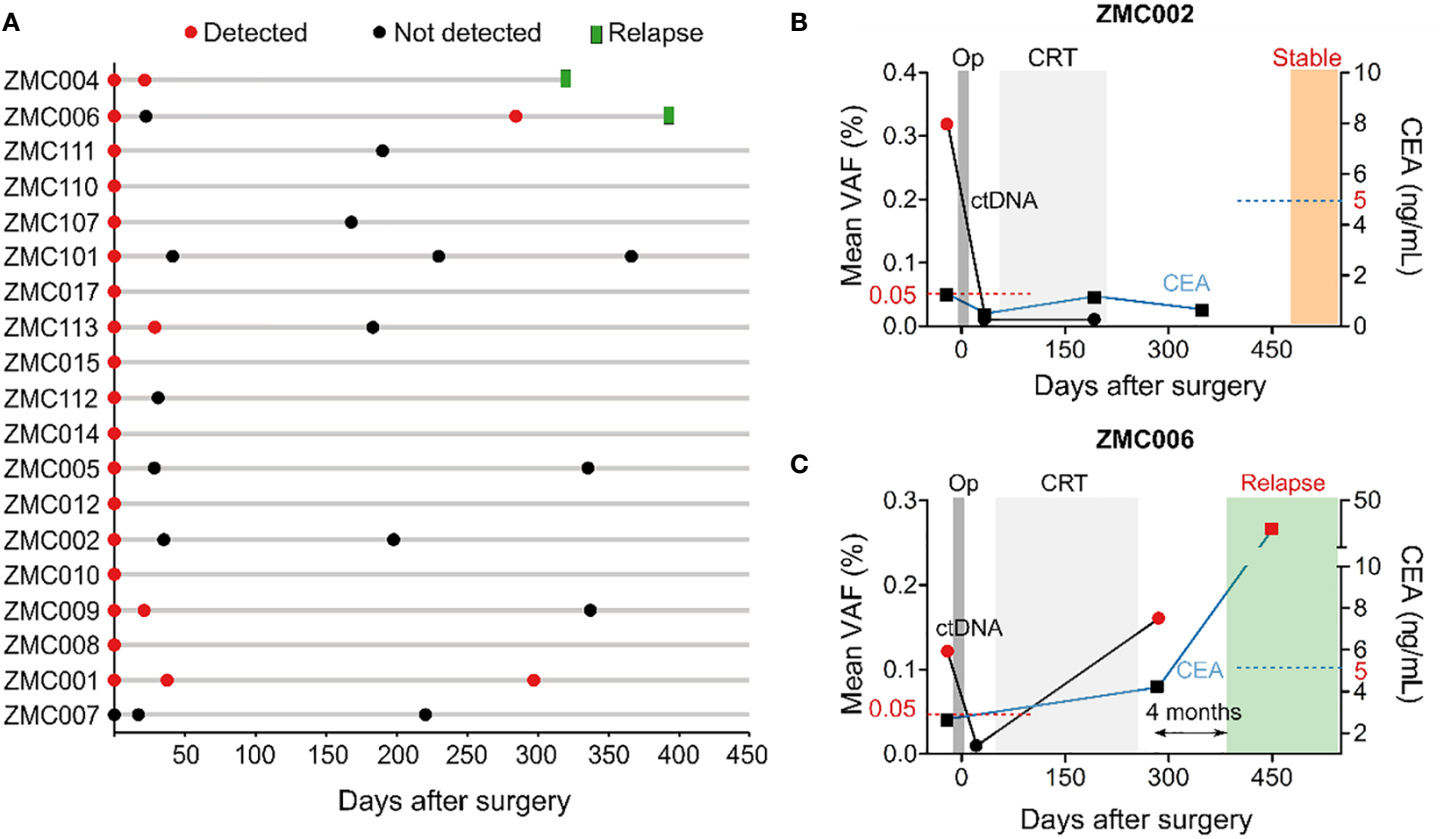
Longitudinal monitoring of ctDNA and clinical outcomes of patients. **(A)** Swimmer plot depicting ctDNA results over time and incidence of relapse in 19 patients that had been followed up for at least 16 months. This was an interim analysis as the study is on-going. **(B, C)** Longitudinal plot showing the mean VAF of ctDNA, CEA level, treatment and clinical status over time of patients ZMC002 and ZMC006. Molecular relapse detection was 4 months earlier than clinically diagnosed relapse in patient ZMC006. CEA level was still normal at the time point when ctDNA was found positive. Op, operation, CRT, chemoradiotherapy.

We next compared the dynamics of cfDNA, ctDNA, and CEA levels after surgery. The results showed that total level of cfDNA was not different between pre-operative and post-operative samples. Meanwhile, the ctDNA level, measured as the mean VAF of the tracked mutations, and the CEA level significantly reduced after surgery, correlating with the clinical removal of tumor burden (**Figure 4E**). The result of ctDNA clearance was then compared with the clinical outcomes of patients who had been followed up for at least 16 months. Out of 19 patients, two were diagnosed with relapse and both of them had ctDNA detected in the plasma 4.0 and 10.5 months earlier than clinical diagnosis (**Figure 5A**). Two case studies were illustrated in more detail. Patient ZMC002 with stage II colon cancer had pre-operative ctDNA(+) but normal CEA level; after surgery, ctDNA was undetected in all follow-up plasma samples, aligning with the clinical evaluation of full remission (**Figure 5B**). Patient ZMC006 also with stage II colon cancer, had ctDNA detected in the plasma sample at 6 months after surgery but was clinically stable at that point. He was later diagnosed with liver and lung metastasis at 10 months after surgery by CT scan. CEA level remained normal both before surgery and at the time point when ctDNA was positive (**Figure 5C**).

## Discussion

In this study, we generated the first somatic variant dataset for Vietnamese CRC patients and evaluated the clinical actionability of the alterations. Using our panel of 95 cancer-associated genes, we found that the mutational burden varied greatly among patients (0-237 mutations), with an average of 7 mutations per patient. This data is consistent with the reported wide range of tumor mutational burden in CRC and also suggested that some hypermutated cases in our cohort could have microsatellite instability (23).

The most frequently mutated genes in our Vietnamese cohort were *APC, TP53* and *KRAS*, agreeing with the well documented data in other Asian and Caucasian cohorts (17–21). *FAT4* was among the top mutated genes in CRC but our mutation frequency seemed to be higher than the Asian (20, 24) and more similar to the Caucasian. *FAT4* mutations were reported to have good prognosis and be a predictive biomarker for better response to immunotherapy (25, 26). Furthermore, our mutual exclusivity analysis showed several major driver genes such as *TP53* with *KRAS*, *TP53* with *PIK3CA*, similar to several other cohorts (17, 18, 21), but not *APC* and *PIK3CA* as reported in the Taiwanese (24). This result could be affected by the gene panel used and the sample number in different studies, but might also suggest potential discrepancy in the carcinogenic pathways among different ethnicities.

Our data showed that up to 41.7% of the Vietnamese patients harbored a resistance mutation in either *KRAS* or *NRAS* that could affect their response to Cetuximab and Panitumumab respectively. This result strongly highlights the necessity of comprehensive genetic analysis to help physicians select appropriate treatment plan for individual CRC patient. Moreover, Wnt/β-catenin, genome integrity and MAPK signaling were found the most commonly altered pathways in our cohort, similar to previous reports (27). There are currently a few experimental drugs in both clinical studies and laboratory research (**Table S2**) targeting alterations in the MAPK signaling in CRC. This could hopefully translate to future access to more tailored therapies for CRC patients.

Our K-Track^®^ assay utilized tumor-derived mutations in 95 genes to design a personalized 5-plex mPCR assay to detect ctDNA in liquid biopsy. This approach is fairly simplified compared to multiple studies using tumor whole exome sequencing and mPCR for 16 amplicons (**Table S3**). Using a small gene panel focusing only on strong cancer-associated genes has advantages of lower background noise, reduced data workload and lower sequencing cost compared to whole exome sequencing. This ultimately makes the assay more high-throughput and affordable for routine testing in Vietnam and probably other developing countries. Interestingly, although reducing the number of mutations to track was reported to modestly compromise the sensitivity of the assay (28), a recent report from Henriksen et al. argued that tracking 1 mutation was as sensitive as 16 mutations in CRC relapse detection (29). In this study, despite using a small gene panel, we detected somatic mutations in 99.0% of patients. The analytical validation of K-Track^®^ mPCR NGS platform allowed for the limit of detection at 0.05% and the specificity of > 99%. This LOD is lower than a few platforms achieving LOD at 0.01% (28, 30) but outperformed several others with LOD of ≥ 0.1% (31–33).

The pre-operative ctDNA detection rate for all patients was 90.5%, higher than the 63.8-74.0% rates in similar assays using gene panels (8, 34, 35); and comparable to the 88.5-96.0% rates in studies using whole exome sequencing approach (7, 29, 36, 37) (details in **Table S3**). The non-inferior performance of our K-Track^®^ again supported both the clinical and economic values of the assay. Furthermore, consistent with previous publications (7, 36), we observed that TNM stage was associated with the pre-operative ctDNA detection rate, that stage I tumors seemed to release less ctDNA into the bloodstream than the stage II-IV tumors. CEA, the primary biomarker for CRC, had fairly low pre-operative detection rate of only 41.3%, as also reported previously (7, 35). Even in patients with elevated CEA level before surgery, the drop in CEA level following total tumor excision was less pronounced than that in ctDNA. Therefore, we conclude that ctDNA appeared to be a more sensitive and reliable signal than CEA to reflect the dynamics of tumor burden.

After 16-month follow up, 2 cases that were clinically diagnosed with metastasis or relapse had post-operative ctDNA(+) with the lead time of 4-10.5 months, comparable with the median lead time of 4-11.5 months in other assays (**Table S3**). Meanwhile, in both patients who relapsed, the CEA level remained normal at the time points when ctDNA was positive. Our findings agreed with Reinert et al. (7) that ctDNA could be a more effective monitoring tool than CEA for CRC patients during post-operative surveillance.

The major limitation of this report was that the clinical data was not yet mature as the study is on-going. A more comprehensive assessment to conclude the sensitivity and specificity of the K-Track^®^ assay in relapse detection is warranted upon study completion. Besides that, the current design for K-Track^®^ assay was tumor-guided, making its accuracy highly dependent on tumor sample availability, FFPE quality and sampling location. A blood-only design that bypasses tumor requirement appears to be more convenient, and has been shown to achieve comparable accuracy with tumor-guided approach in CRC patients (3, 38). We are investigating the feasibility of this approach both technically and economically as these studies also had to combine assays on epigenomic features together with mutations to identify ctDNA (3, 38).

In conclusion, we provided the first somatic variant landscape of the Vietnamese CRC patients that contributes to the knowledge base of the genetic complexity of colorectal cancer. We also developed a streamlined K-Track^®^ assay that showed promising dual clinical utilities in residual cancer surveillance and actionable mutation profiling for targeted therapies. Although the performance of the assay needs to be fully evaluated after study completion, this report supports that K-Track^®^ could be the affordable approach to precision oncology in Vietnam and possibly other developing countries.

## Data availability statement

The data presented in the study are deposited in the BioProject repository, accession number PRJNA902849 https://www.ncbi.nlm.nih.gov/bioproject/PRJNA902849.

## Ethics statement

The studies involving human participants were reviewed and approved by the institutional ethics committees of the Thu Duc city Hospital (approval number 17/HDDD) and the University of Medicine and Pharmacy, Ho Chi Minh city (approval number 14/GCN-HDDD for the study at University Medical Center and approval number 164/HDDD for the study at the Medical Genetics Institute). The patients/participants provided their written informed consent to participate in this study.

## Author contributions

HN, TVN, DT, NT, ML, TP, TD, TSN, KT, HM, MC, DP, XN, DSN, DQN, Y-TL, QN recruited patients and performed clinical analysis. V-AH, T-AT, TH, TT, DT, H-NN, HG processed samples and analyzed genetic data. LT designed experiments, analyzed data and wrote the manuscript. All authors contributed to the article and approved the submitted version.
