# Supplementary material for: Tumor genomic profiling and personalized tracking of circulating tumor DNA in Vietnamese colorectal cancer patients
Source: Front Oncol. 2022 Dec 12;12:1069296. doi: 10.3389/fonc.2022.1069296 (PMC9792166; doi:10.3389/fonc.2022.1069296)
Supplement: Supplementary file 1 [file DataSheet_1.pdf]

Table S1. List of 95 targeted genes

ACVR2A	*CDKN2A*	*GATA3*	*NFE2L2*	*SETD2*
AFF3	*CREBBP*	*GNAS*	*NOTCH1*	*SMAD4*
AKT1	*CTNNB1*	*GPC3*	*NOTCH2*	*SMARCA4*
ALK	*DDR2*	*GPHN*	*NRAS*	*SPOP*
AMER1	*DICER1*	*GRIN2A*	*NSD1*	*STAG2*
APC	*DNMT3A*	*HLA-A*	*NTRK3*	*STK11*
AR	*EGFR*	*HRAS*	*PDE4DIP*	*TBX3*
ARID1A	*EP300*	*IDH1*	*PIK3CA*	*TCF7L2*
ARID1B	*ERBB2*	*KDM6A*	*PIK3R1*	*TP53*
ARID2	*ERBB3*	*KEAP1*	*PREX2*	*TRRAP*
ATM	*ERBB4*	*KMT2A*	*PTEN*	*TSC1*
ATR	*ERCC2*	*KMT2C*	*PTPN13*	*TSC2*
AXIN1	*ESR1*	*KMT2D*	*PTPRB*	*TSHR*
BCOR	*FAT1*	*KRAS*	*PTPRT*	*ZFHX3*
BRAF	*FAT4*	*LRP1B*	*RAD51B*	*ZNF521*
BRCA1	*FBXW7*	*MAP3K1*	*RB1*	
BRCA2	*FGFR3*	*MET*	*RBM10*	
CAMTA1	*FHIT*	*NCOR1*	*RNF213*	
CASP8	*FOXA1*	*NCOR2*	*RNF43*	
CDH1	*FOXP1*	*NF1*	*RSPO2*	

Table S2. Actionable alterations and OncoKB™ therapeutic level of evidence

Gene	Alterations	Drug	Level*
BRAF	V600E	Encorafenib + Cetuximab	1
KRAS	Wild type	Cetuximab<br>Cetuximab + Chemotherapy<br>Panitumumab<br>Panitumumab + Chemotherapy	1
NRAS	Wild type	Panitumumab<br>Panitumumab + Chemotherapy	1
NTRK3	Fusions	Entrectinib, Larotrectinib	1
BRAF	V600E	Encorafenib + Panitumumab	2
ERBB2	Amplification	Lapatinib + Trastuzumab<br>Trastuzumab + Pertuzumab<br>Trastuzumab Deruxtecan	2
KRAS	G12C	Adagrasib<br>Adagrasib + Cetuximab	3
NTRK3	Fusions	Repotrectinib	3
ARID1A	Truncating Mutations	PLX2853, Tazemetostat	4
BRAF	G464, G469A, G469R, G469V, K601, L597	PLX8394	4
CDKN2A	Oncogenic Mutations	Palbociclib, Ribociclib, Abemaciclib	4
FGFR3	Oncogenic Mutations	Debio1347, Infigratinib, Erdafitinib, AZD4547	4
KRAS	Oncogenic Mutations	Trametinib, Cobimetinib, Binimetinib	4
MET	Fusions	Crizotinib	4
NF1	Oncogenic Mutations	Trametinib, Cobimetinib	4
PTEN	Oncogenic Mutations	GSK2636771, AZD8186	4
KRAS	Oncogenic Mutations	Cetuximab, Panitumumab	R1
NRAS	Oncogenic Mutations	Cetuximab, Panitumumab	R1
NTRK3	F617L, G623R, G696A	Larotrectinib	R1

* Level 1: FDA-recognized biomarker for FDA-approved drugs
Level 2: Standard care biomarker recommended by professional guidelines for FDA-approved drugs
Level 3: Compelling clinical evidence supports the biomarker for a drug
Level 4: Compelling biological evidence supports the biomarker for a drug
Level R1: Standard care biomarker predictive of resistance to FDA-approved drugs

Table S3. Design and results of different MRD assays in colorectal cancer

Studies	Cohort	Stage	Tumor sequencing	ctDNA tracking	Pre-op ctDNA detection	Relapse detection
**Tie et al, 2019 (8)**	159	Locally advanced	15 genes	1 mutation	77%	Sens 48%<br>Spec 94%
**Tarazona et al, 2019 (33)**	150	I-III	29 genes	≥2 mutations	63.8%	Sens 87.5%<br>LT 11.5 months
**Schøler et al, 2017 (34)**	45	I-III	WES	≥1 mutation	74%	Sens 100%<br>Spec 100%<br>LT 9.4 months
**Reinert et al, 2019 (7)**	125	I-III	WES	16 mutations	88.5%<br>Stage I: 40%<br>Stage II: 92%<br>Stage III: 90%	Sens 88%<br>Spec 98%<br>LT 8.7 months
**Shirasu et al, 2021 (35)**	1236	I-III	WES	16 mutations	92%<br>Stage I: 80%<br>Stage II: 96%<br>Stage III: 94%	
**Henriksen et al, 2022 (28)**	112	II-III	WES	1 mutation vs. 16 mutations	82% (1 mutation), 96% (16 mutations)	Sens 45-50%<br>Spec 94%<br>LT 4 months
**Henriksen et al, 2022 (36)**	160	III	WES	16 mutations	91%	LT 9.8 months
**Vidal et al, 2021 (37)**	72	II-IV	No	Mutation + Methylation	83%	
**Parikh et al, 2021 (3)**	103	I-IV	No	Mutation + Methylation	-	Sens 91%<br>Spec 100%<br>LT 10.9 months

* Sens = sensitivity. Spec = specificity. LT = Lead time

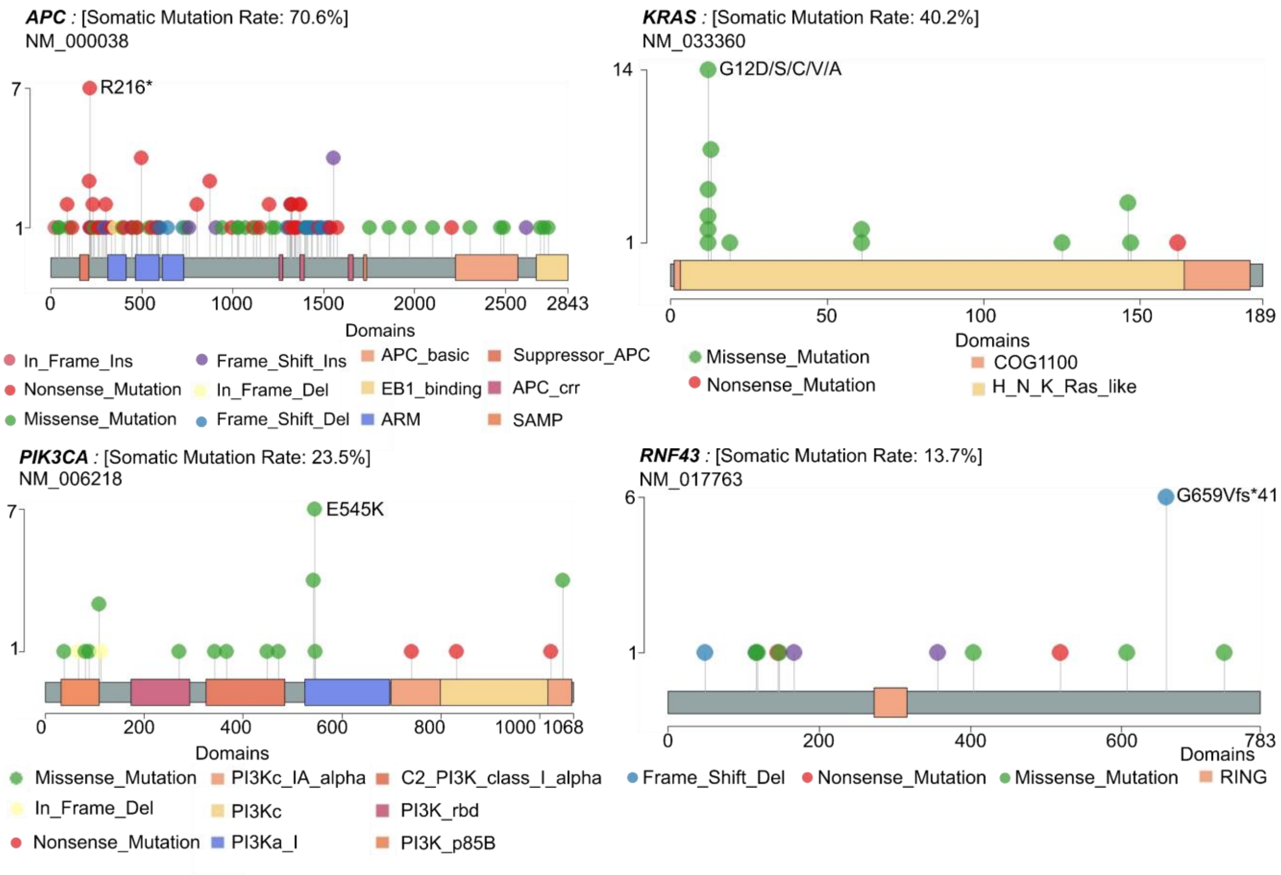

Figure S1. Hotspot mutations in top mutated genes. Lollipop plots displaying the mutation distribution across protein domains for *APC, KRAS, PIK3CA* and *RNF43*. *APC* R216*, *KRAS* G12, *PIK3CA* E545 and *RNF43* G659Vfs*41 were the hotspot recurrent mutations in the Vietnamese colorectal cancer patients.

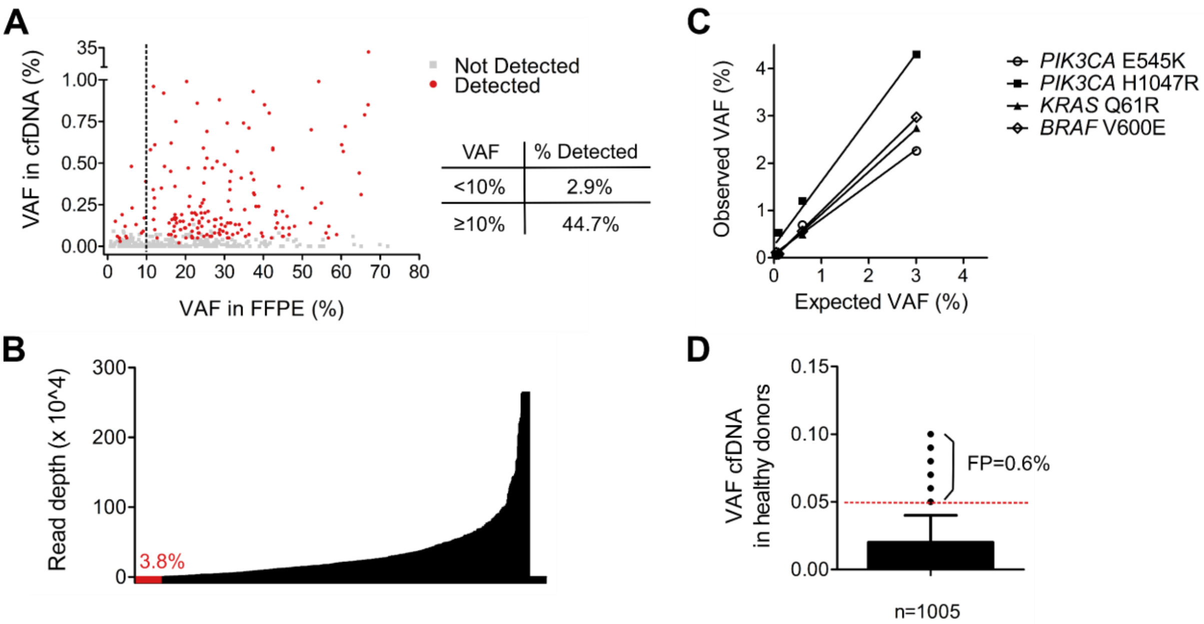

Figure S2. Analytical performance of ctDNA detection assay. (A) There was no correlation between VAF of a mutation in FFPE and its VAF in plasma. However, mutations with VAF in FFPE ≥ 10% had higher detection rate in plasma compared to those with VAF in FFPE < 10%. **(B)** The distribution of read depth per amplicon in all analyzed plasma samples. 3.8% of the amplicons with sequencing coverage less than 10,000X (marked red) were excluded from downstream analysis. **(C)** Graph of the titration series for *PIK3CA* E545K, *PIK3CA* H1047R, *KRAS* Q61R, and *BRAF* V600E to determine limit of detection at 0.05%. **(D)** The false-positive (FP) rate was determined using plasma samples from 150 healthy donors. Analysis of 1005 amplicons showed FP rate at 0.6%.
